# New genus and species of flea beetles (Coleoptera, Chrysomelidae, Galerucinae, Alticini) from Puerto Rico, with comments on flea beetle diversity in the West Indies and a key to the West Indian Monoplatini genera

**DOI:** 10.3897/zookeys.155.2124

**Published:** 2011-12-15

**Authors:** A. S. Konstantinov, A. A. Konstantinova

**Affiliations:** 1 (ASK) Systematic Entomology Laboratory, PSI, Agricultural Research Service, U.S. Department of Agriculture, c/o Smithsonian Institution, P.O. Box 37012, National Museum of Natural History, MRC 168 Washington, DC 20013-7012, USA; 2 (AAK) Franklin W. Olin College of Engineering, Olin Way, Needham, MA 02492 USA

**Keywords:** Leaf beetles, species diversity, moss, West Indies, Puerto Rico

## Abstract

A new genus (*Borinken*) and five new species (*Borinken elyunque*, *Distigmoptera chamorrae*, *Kiskeya elyunque*, *Ulrica eltoro*, and *Ulrica iviei*) from Puerto Rico are described and illustrated. A keyto all West Indian Monoplatini genera is provided, as are keys to all speciesof *Kiskeya* and to the speciesof *Ulrica* from Puerto Rico. A list of the flea beetle genera, along with the number of species and some of the faunal features is presented and discussed for the West Indies.

## Introduction

The West Indies Islands are one of the World’s biodiversity hotspots (Myers et al. 2000). A great variety of ecosystems exist in the West Indies, ranging from tropical moist broadleaf forests to xeric cactus scrublands. The West Indian flora and fauna are rich and highly endemic. Seventy-two percent of 11,000 plant species of the West Indies are endemic (BirdLife International 2009). Among vertebrates, 99% of amphibians and 93% of reptiles are endemic (Hedges 2001). To date, 351 species of flea beetles in 53 genera are known to occur in the West Indies (Table 1). Compared to other regions of the World, these relatively small land masses are home to considerable species richness. For example, a much larger territory of the European part of the former U.S.S.R. (stretching from the White Sea on the North to the Black Sea on the South, including the Caucasus) has 321 species in 24 genera of flea beetles; none of the genera are endemic, and only 20% of the species are endemic (Konstantinov 1991). In the West Indies, 92% of flea beetle species and 20% of genera (marked bold in the Table 1) are endemic. In addition, only 13% of species occur on more than one island. *Epitrix parvula* Fabricius is the most widespread flea beetle species, being found on most West Indian Islands and in North and Central America. While the number of flea beetle species in the European part of the former U.S.S.R., including the Caucasus, and the West Indies are similar (321 and 351 respectively), the number of genera in the West Indies is more than twice that in the European part of the former U.S.S.R. and the Caucasus, which makes the fauna of the significant part of the Palearctic more of an “island” fauna than the island fauna itself (Konstantinov et al. 2009). As a rule, an island fauna is characterized by a relatively large number of species per higher taxon as a result of a relatively small number of introductions followed by extensive species-level radiation (Magnacca and Danford 2006). The fauna of the European part of the former U.S.S.R. (being part of the Palearctic) was recently dramatically changed by the Tertiary aridization and Quaternary glaciation (Konstantinov et al. 2009), while the West Indian fauna remained relatively intact.

The flea beetles of the West Indies are comparatively well studied. Extensive collecting and publications during the first half of the 20^th^ and early 21^st^ century reported many unusual and endemic flea beetle taxa (Blake 1928, 1931, 1934, 1937, 1938, 1944, 1947, 1960, 1964, Konstantinov 2002, Konstantinov and Chamorro-Lacayo 2006). The three most species rich genera in the West Indies are *Aedmon* Clark with 36 species, *Homoschema* Blake with 26 species, and *Monomacra* Chevrolat with 22 species. Two of these most speciose genera are West Indian endemics. The distribution of species in the endemic genera reveals some aspects of the faunistic relationships between different islands (Table 1). For example, the moss-inhabiting *Kiskeya* Konstantinov and Chamorro-Lacayo has three species in the West Indies, two in the Dominican Republic and one in Puerto Rico. *Kiskeya* is morphologically similar to the Oriental *Clavicornaltica* Scherer, alluding to broader biogeographical patterns of moss and leaf litter inhabiting flea beetles. *Normaltica* Konstantinov has a similar pattern with one species in the Dominican Republic and one in Puerto Rico. The apparent absence of *Kiskeya* and *Normaltica* in Cuba probably reflects more a lack of collecting in that largest West Indian Island than to actual biogeographical patterns. Three currently known species of *Monotalla* Bechyné (two of which are undescribed) occur only in the Lesser Antilles. The only two species of *Bonfilsus* Scherer occur in Hispaniola and Guadeloupe (one species per island).

Recent collecting efforts in Puerto Rico revealed unique flea beetles with several features rarely observed among flea beetle genera.

**Table 1.** Flea beetle species diversity in the West Indies (we grouped some islands in a few columns because there are just a few species known to occur there. Names in bold indicate that taxon is endemic for West Indies. To make this table current, we include two undescribed species of *Monotalla* from Dominica and St. Lucia, manuscript describing them is in preparation).

**Genus Author**	**Cuba**	**Hispa-niola**	**Jamaica**	**Puerto Rico**	**Grenada**	**Baha-mas**	**Antigua & VirginIslands Barbuda St. Lucia Montserrat**	**Guade-loupe**	**Dominica**	**St. Croix St. Thomas**	**St. Vincent**	**Total species**
*Acallepitrix* Bechyné								3				3
*Acrocyum* Jacoby		1										1
***Aedmon*** Clark		21		8				2	6			36
*Alagoasa* Bechyné	1	4									1	6
*Altica* Geoffroy	5				1						1	6
***Apleuraltica*** Bechyné								1				1
***Apraea*** Jacoby	8	4	4	3		1						19
*Argopistes* Motschulsky	1	1		1		1						4
*Asphaera* Chevrolat	1											1
*Blepharida* Chevrolat				1								1
***Bonfilsus*** Scherer		1						1				2
***Borinken***Konstantinov & Konstantinova				1								1
*Centralaphthona* Bechyné	5	3	1	6	2						2	16
*Chaetocnema* Stephens	5	5	4	5	2	1		2		1	1	16
*Cyrsylus* Jacoby	1	1	1	1			1			2		5
*Diphaulaca* Chevrolat	?	?		?						1		2
*Disonycha* Chevrolat	7	3	4	4	3							13
*Distigmoptera* Blake		1		1								2
*Epitrix* Foudras	3			3	3						1	5
*Exoceras* Jacoby								1			1	2
*Gioia* Bechyne	1		3					3	1			8
*Glyptina* LeConte					1							1
***Guadeloupena*** Bechyné								1				1
*Heikertingerella* Csiki	2	2	1	1	1			5	2		1	13
*Hemilactica* Blake	7	1		1								9
***Hirtiasphaera*** Medvedev		1										1
***Homoschema*** Blake	6	5	4	5		2		1	2	1		26
*Homotyphus* Clark								1				1
*Hypolampsis* Clark				1	1						1	2
***Kiskeya*** Konstantinov & Chamorro-Lacayo		2		1								3
*Kuschelina* Bechyné						1						1
*Leptophysa* Baly	3	4	2	1				1				11
*Longitarsus* Latreille	7	2	1	6	1	3	2	1			1	21
*Lupraea* Jacoby		1						1				2
*Lysathia* Bechyné	1	1	1	2			2		1			2
*Macrohaltica* Bechyné	1	2	1	1								2
*Megasus* Jacoby			1									1
*Megistops* Boheman	4	2	2	3	1	1		1				12
*Monomacra* Chevrolat	3	1	6	1	2	3		2	3		2	22
***Monotalla*** Bechyné							1	1	1			3
*Neothona* Bechyné	2		1	2								1
*Nesaecrepida* Blake	2		2	1								2
***Normaltica*** Konstantinov		1		1								2
*Oedionychus* Berthold	12	1	2		1						1	17
*Omophoita* Chevrolat	2	2	1	2	1		1	1		2	1	5
*Phyllotreta* Chevrolat	1		1	2								3
*Physimerus* Clark					1						1	1
*Platiprosopus* Chevrolat	?	?		?				2				2
*Pseudodisonycha* Blake	2	1		1								4
*Strabala* Chevrolat	5	1	2	2								7
*Syphraea* Baly	1	3	4	2	1	1		1			1	14
*Systena* Chevrolat	3	1	2	2	2						2	7
*Ulrica* Scherer				2								2
Number of genera/species	30/102	32/79	23/51	33/74	16/24	9/14	5/7	20/32	7/16	5/7	15/18	53/351

## Materials and methods

Various collecting methods were used in Puerto Rico. Among them, beetles from sifted and unsifted moss samples by Berlese extraction represent most of the new taxa described here. Collecting in moss cushions was one of the most effective methods for uncovering a previously unknown fauna.

Dissecting techniques and terminology for most internal and external structures follow Konstantinov (1998, 2002). Terminology for the thoracic structures follows Konstantinov and Lopatin (1987) and Chamorro-Lacayo and Konstantinov (2004). We follow a format in which we provide a detailed description of a genus with relatively short species descriptions mentioning characters that are useful for species separation. For the new species of *Distigmoptera* Blake, the description is relatively long and contains characters that are helpful in determining generic placement given uncertainty in the generic classification of Monoplatini. The beetles are deposited in the following collections: National Museum of Natural History, Smithsonian Institution, Washington, D.C. (USNM); West Indian Beetle Fauna Project Collection, Montana State University, Bozeman, Montana (WIBF); and Monte L. Bean Life Science Museum, Brigham Young University, Provo, Utah (MLBU).

### Borinken

Konstantinov & Konstantinova gen. n.

urn:lsid:zoobank.org:act:B5FCB199-B009-47CD-960D-76ACAE936BFD

http://species-id.net/wiki/Borinken

Figs 1 [Fig F2] –14

#### Description.

Body length 1.08–1.18 mm, width 0.70–0.81 mm, elongate, relatively flat in lateral view (2.18 times as long as thick). Color brown without metallic luster, legs slightly lighter and antennae, except last antennomere, darker, almost black.

Head (Figs 4, 5) flat in lateral view. Frons and vertex forming nearly straight line (Fig. 5) in lateral view. Facial part of head elongate. Supraorbital pore situated near outer corner of antennal callus, poorly visible. Antennal calli well developed, slightly longer than wide, oblique, separated from each other by wide midfrontal sulcus. Supracallinal sulcus deep, convex. Suprafrontal and supraantennal sulci well developed, deep. Supraorbital sulcus slightly impressed. Orbit as wide as transverse diameter of eye. Interantennal space nearly as wide as transverse diameter of eye and as transverse diameter of antennal socket. Frontal ridge narrow, lowering in front of anterofrontal ridge. Anterofrontal ridge not separated from frontal ridge, long and swollen. Two ridges situated laterally of frontal ridge from lower margin of antennal socket to outer corner of mouth. Long seta situated at beginning of each ridge under antennal socket. Another long seta located on both sides of frontal ridge. Eyes small, slightly protruding laterally, 0.72 times as wide as long. Vertex covered with few large and deep punctures. Labrum with six setiferous pores, apically slightly incised. Labium with three palpomeres per palpus, distal palpomere longer than wide (Fig. 6). Maxillary palpus with four palpomeres, distal palpomere conical, slightly longer than preapical, sensilla patch with three setae (Fig. 8). Antenna with 11 antennomeres. First antennomere slightly wider and much longer than second and rest of antennomeres separately. Third and fourth antennomeres much thinner than second. Antennomeres gradually widening distally (Fig. 7).

**Figures 1–5.**
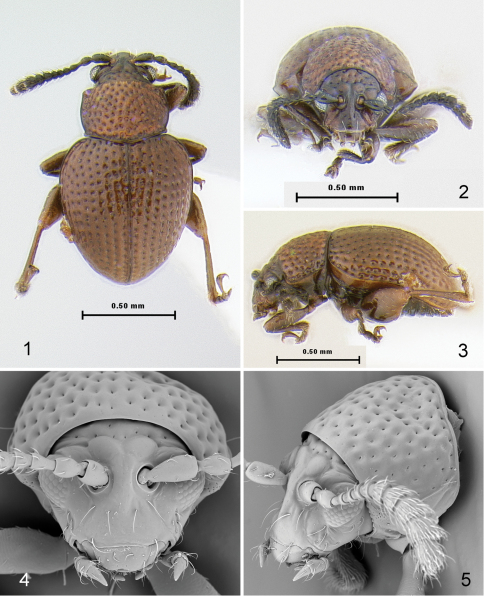
*Borinken elyunque*: **1** habitus, dorsal view **2** habitus, frontalview **3** habitus, lateral view **4** head and pronotum, frontal view **5** head and pronotum, lateral view.

Pronotum (Fig. 1) 1.34 times wider than long (measured in middle), without impressions, covered with large, deeply impressed punctures. Sides weakly rounded and relatively narrowly explanate, with maximum width in front of middle. Marginal anterolateral callosity situated perpendicularly to midline of body, 3.71 times shorter than lateral margin. Posterolateral callosity protruding laterally. Basal margin evenly convex, slightly extends posteriorly, with distinct border. Procoxal cavity open. Intercoxal prosternal process relatively narrow and parallel-sided in middle, with longitudinal ridge bordered by two deep grooves laterally, abruptly expanding beyond procoxae. Scutellum flat, wider than long, apex sharply triangular, sides straight. Mesocoxae separated by both meso- and metasterna. Mesosternum not covered by metasternum, horizontal (Fig. 9). Metasternum (Fig. 9) protruding anteriorly between mesocoxae, wide, nearly flat at apex.

Elytron (Fig. 1) widest near mid-length. Humeral callus absent. Elytral punctures arranged in nine rows not counting scutellar row. Punctures large, about as large as space between rows. Elytral apex narrowly rounded, surrounded by distinct border. Epipleura broad, slightly oblique, gradually narrowing posteriorly, not attaining sutural margin of elytron. Elytron with sensilla distributed evenly throughout surface, others concentrated in single sensilla patches (Fig. 14). Elytra fused. Elytral lock consists of longitudinal groove along its suture (Fig. 14). Wings absent.

Pro- and mesofemora normally round, only slightly flat dorsoventrally. Metafemur robust, flat dorsoventrally, fairly symmetrical (Fig. 10), 2.15 times as long as wide. Pro- and mesotibiae cylindrical, slightly wider in distal 1/3, without spurs apically. Metatibia (Figs 1, 9) straight in dorsal and lateral views, generally cylindrical, gradually widening distally (in dorsal view), dorsal surface convex nearly to apex. Apical spur long, slightly shorter than tarsal claw. Claw appendiculate near base. Third tarsomere deeply incised (Fig. 11). First metatarsomere as long as two following tarsomeres together.

Abdomen with five distinctly visible sternites. Apical sternite shorter than three preceding sternites combined, without appendages basally. Basal sternite longer than four following sternites together.

Median lobe of aedeagus (Fig. 12) simple, robust, slightly and evenly curved in lateral view, without any sculpture ventrally.

#### Type species.

*Borinken elyunque* Konstantinov & Konstantinova, new species.

#### Etymology.

This genus is based on the native Taino Indian name for the Island of Puerto Rico, *Borinquen*. The name is masculine.

#### Diagnosis and comparison.

*Borinken* is very different from other flea beetle genera that are known to occur in mosses in the New World (*Kiskeya* in the West Indies, *Nicaltica* Konstantinov, Chamorro-Lacayo and Savini in Nicaragua, and *Ulrica* Scherer in the West Indies and Central and South America). Based on the general shape of the body, shape of the base of the pronotum without a lobe extending posteriorly, general shape of the metatibia and tarsal claw, *Borinken* is similar to *Benedictus* Scherer, which inhabits mosses in Asia and does not occur in the New World (Sprecher-Uebersax et al. 2009). It can be easily distinguished from that genus by the unique shape and details of the head, subquadrate apical antennomeres, and absence of the prebasal impression on the pronotum.

*Borinken* is also very different from any other West Indian or New World flea beetle genera. Among New World genera it is somewhat similar to *Centralaphthona* Bechyné based on the presence of antennal calli, lack of the prebasal groove on the pronotum, regular elytral striae and open procoxal cavities. *Borinken* can be easily differentiated from *Centralaphthona* by the following features: elongate facial part of head (normally short in *Centralaphthona*); antennal calli longer than wide (usually shorter than wide in *Centralaphthona*); apical antennomeres much wider than basal (about same width in *Centralaphthona*); vertex, pronotum, and elytra strongly punctured (punctation normally small in *Centralaphthona*; overall, this kind of coarse punctation is rare among flea beetles); apex of metatibia convex up to tarsomere (flat in *Centralaphthona*).

**Figures 6–11. F2:**
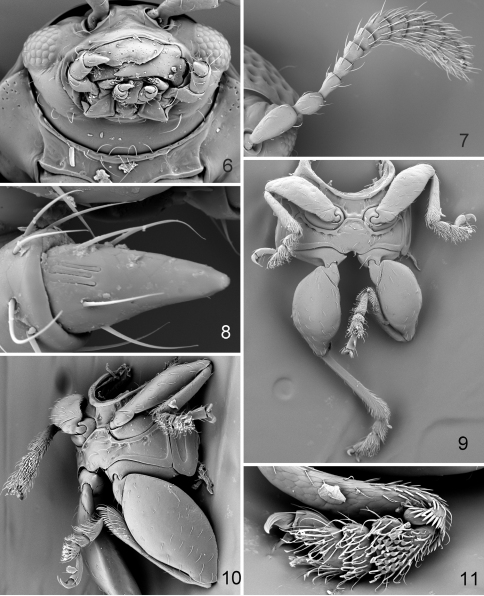
*Borinken elyunque*: **6** head, anteroventral view **7** antenna **8** last maxillary palpomere **9** thoracic sternites with mid- and hind legs, ventral view **10** thoracic sternites with mid- and hind legs, lateral view **11** protibia and protarsus.

**Figures 12–14.**
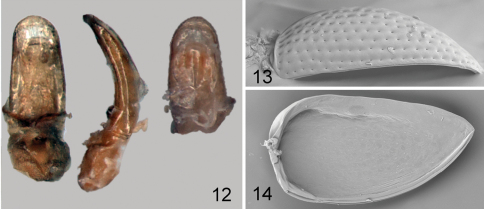
*Borinken elyunque*: **12** median lobe of aedeagus, ventral, lateral and dorsal views **13** right elytron, lateral view **14** left elytron, ventral view.

### Borinken elyunque

Konstantinov & Konstantinova sp. n.

urn:lsid:zoobank.org:act:30A86614-5362-4AE0-9372-80B07A98BBD4

http://species-id.net/wiki/Borinken_elyunque

Figs 1 [Fig F2] –14

#### Description.

Body length 1.08–1.18 mm, width 0.70–0.81 mm, elongate, relatively flat in lateral view (2.18 times as long as thick). Color brown without metallic luster, legs slightly lighter and antennae, except last antennomere, darker, almost black. Vertex covered with large punctures, shiny, without wrinkles. Oblique fold situated between orbit and antennal callus. Proportions of antennomere lengths in male: 14:9:6:6:6:4:5:5:5:7:10. Antennomeres widened abruptly beginning from antennomere 7 (it is 0.71 times as long as wide). Pronotum evenly covered with large punctures, their diameter much larger than distance between them. Ventral side of body without many setae. Elytron with nine complete rows of punctures. Additional scutellar row incomplete. Punctures large, about as large as space between rows. Interspaces shiny with wrinkles or punctures. Proportions of tarsomere lengths of male as follows: protarsomeres 5:4:4:11; mesotarsomeres 5:4:4:11; metatarsomeres 9:4:5:11.

Median lobe of aedeagus (Fig. 12) robust in ventral view, with apex evenly convex without apical denticle. Apex slightly swollen in lateral view. Ventral side flat, without membranous window.

#### Etymology.

The specific epithet is a noun in apposition based on the type locality.

#### Ecology.

Unidentified moss samples that contained *Borinken elyunque* were collected in the forest from a variety of substrates (rocks, tree stumps, trunks and branches) (Figs 59, 60).

#### Type material.

Holotype: ♂, Puerto Rico: El Yunque, El Toro trail, 18°16.850'N, 65°49.753W, 1066m, 14.VI.2008, moss (unsifted) leg. A. Konstantinov (USNM). Paratype ♂, same label as holotype (USNM). Paratypes 2 ♂, same label as holotype except the date, 16.VI.2008 and moss being “unsifted” (USNM).

### Distigmoptera

Blake

http://species-id.net/wiki/Distigmoptera

Figs 15 –27

Distigmoptera Blake, 1943: 209 (type species *Distigmoptera apicalis* Blake, 1943, by original designation).

#### Discussion.

*Distigmoptera* was first recorded in the West Indies by Medvedev (2004) who described a new species from the Dominican Republic. Fourteen previously described species of this genus are known to occur in the USA, Canada, Mexico, and Costa Rica. Among the West Indian genera of Monoplatini, *Distigmoptera* is mostly similar to *Apleuraltica* Bechyné. Apart from characters mentioned in the key (see below), *Distigmoptera* can be differentiated from *Apleuraltica* by the antennae that are not clearly clubbed, antennomere six in males is only slightly different from antennomere seven (the antennae are clearly clubbed, antennomere six in males is markedly different from antennomere seven in being much shorter and narrower than seven in *Apleuraltica*) and by the metatibial apex without a sharp denticle (the metatibial apex has a sharp denticle in *Apleuraltica*).

**Figures 15–21.**
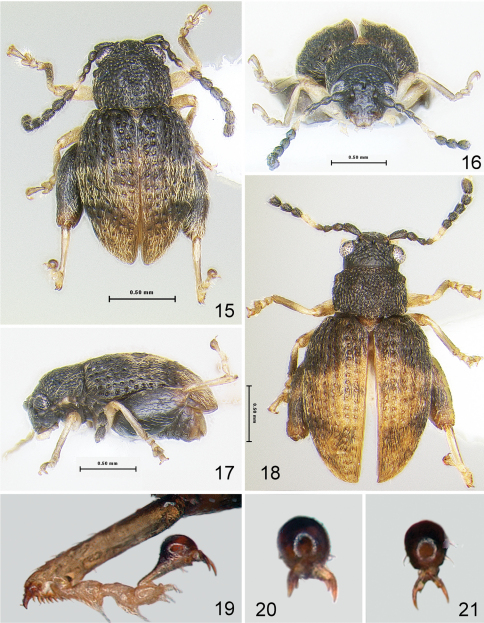
*Distigmoptera chamorrae*: **15** habitus, dorsal view, male **16** habitus, frontalview, female **17** habitus, lateral view, male **18** habitus, dorsal view, female **19** metatibia and metatarsus, lateral view **20** claw, male **21** claw, female.

**Figures 22–27.**
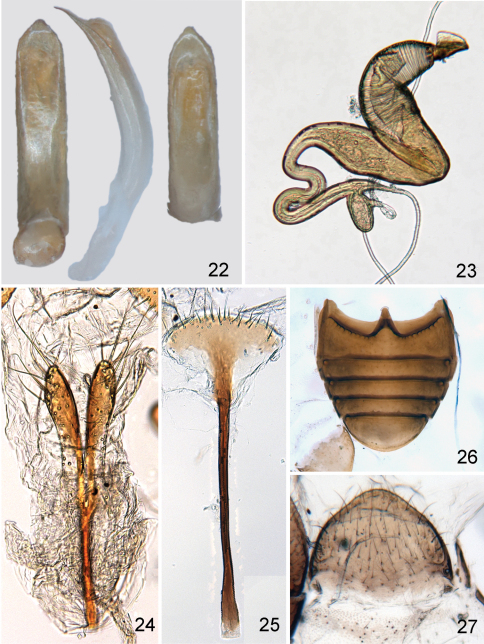
*Distigmoptera chamorrae*: **22** median lobe of aedeagus, ventral, lateral and dorsal views **23** spermatheca **24** vaginal palpi **25** tignum **26** abdominal sternites, female **27** last abdominal tergite.

### Distigmoptera chamorrae

Konstantinov & Konstantinova sp. n.

urn:lsid:zoobank.org:act:3C9F8E9B-A3C3-4264-BC7D-A0CBD4108508

http://species-id.net/wiki/Distigmoptera_chamorrae

Figs 15 –27

#### Description.

Body (Figs 15, 18) length 1.72–2.27 mm, width 0.91–1.14 mm, pubescent, oval and moderately flat in lateral view. Head, except mouthparts, antenna, except antennomere five in males and five and six in females, pronotum, base of elytron, and metafemur dark brown to blackish. Mouthparts, antennomere five in male and five and six in female, front and middle legs and metatibia yellowish to very light brown. Apex of protibia and apical part of elytron (larger in female than in male) slightly darker. Lighter part of elytron with two transverse dark bands, narrower and better separated in female and wider and poorly visible in male. Lighter parts of elytron covered with setae lighter in color, darker parts covered with setae darker in color.

Head (Figs 16, 17) slightly convex in lateral view, evenly and strongly rugose and pubescent. Frons and vertex forming slightly convex line in lateral view. Supraorbital pore indistinguishable. Antennal callus clearly visible, nearly quadrate, its surface situated above surface of vertex. Midfrontal sulcus wide and deep. Supracallinal and supraorbital sulci poorly visible. Suprafrontal and supraantennal sulci shallow. Orbit relatively narrow, 2.50 times narrower than transverse diameter of eye. Interantennal space as wide as transverse diameter of eye. Antennal socket rounded. Frontal ridge wide, parallel sided. Anterofrontal ridge merged with frontal ridge forming denticle in middle. Eyes small, slightly protruding laterally, inner margin curved. Labrum deeply notched in middle, with six setiferous pores. Apical maxillary palpomere as wide as long, conical, much smaller than preceding palpomere. Labial palpomeres of equal length, apical conical. Antenna with 11 antennomeres, antennomeres widening apically. Antennomere four thinnest (Fig. 18). Proportions of antennomere lengths in male: 12:4:6:5:5:5:7:7:7:6:9; in female: 12:6:5:5:5:6:6:6:6:7:9.

Pronotum (Figs 15, 18) 1.47 times wider than long. Pronotal disc anteriorly raised in two wide ridges separated by shallow and wide impressions. Anterior margin straight, with distinct border. Lateral margins subparallel, very slightly convex, without explanation. Posterior margin nearly straight, without distinct border. Anterolateral callosity globular and evenly rounded, bearing seta and not forming denticle posteriorly. Posterolateral callosity absent. Pronotal surface covered with large closely placed punctures and a few yellow, whitish and black setae. Scutellum triangular, densely covered with yellow setae. Prosternal surface densely covered with irregular punctures. Prosternal intercoxal process extended posteriorly beyond coxa and truncate posteriorly. Posterior end about twice as wide as middle. Procoxal cavities closed posteriorly. Mesosternum shorter than prosternal process, quadrate, rugose and pilose. Metasternum smooth and pilose, convex in lateral view, as long as pro- and mesosterna together. Posterior margin with sharp notch.

Elytral surface punctate (Figs 15, 18), with punctures forming nine striae (not counting marginal and short scutellar striae), densely pilose with black setae near base and yellow setae in posterior half. Interspaces between puncture rows two and three, four and five, six and seven form convex ridges. Humeral callus absent. Base of elytron with callus situated between suture and humeral corner. Epipleura wide, nearly vertical, narrowing abruptly at elytral apex but not reaching it. Elytral apex narrowly truncate.

Pro- and mesofemora slightly dilated and tibiae subcylindrical, somewhat enlarged towards apical edge (Fig. 17); pubescence sparsely distributed. Metafemur greatly enlarged, 1.82 times longer than wide and 1.72 times longer than metatibia. Pro- and mesotibiae without apical spurs. Metatibia straight in lateral view, slightly curved in dorsal view. Outer and inner lateral dorsal ridges more or less straight with apical third with numerous denticles. Metatibial spur well developed. First metatarsomere inserted preapically and about as long as two subsequent tarsomeres together. Claw tarsomere swollen. Claw split in male and appendiculate in female.

Abdomen pubescent, with five visible sternites. Apical sternite shorter than three preceding sternites combined, without appendages basally (Fig. 26). Basal sternite shorter than three following sternites together. Last abdominal tergite of female without groove in middle, evenly covered with long setae.

Median lobe simple, slightly curved in lateral view with more abrupt curvature near middle; in ventral view, with lateral margins almost parallel, apex subtriangular without denticle (Fig. 22). Ventral side apically flatter than basally.

In female genitalia, posterior part of sternite eight sclerotized along its entire margin (Fig. 25). Tignum with rounded anterior margin, evenly sclerotized, bearing many moderately long setae (Fig. 25). Vaginal palpi (Fig. 24) elongate, anteriorly and along middle strongly sclerotized and merged anteriorly for more than half of their length, each with about eight setae at apex, with posterior sclerotization shorter than anterior (Fig. 24). Spermatheca curved (Fig. 23), with receptacle and pump not differentiated from each other (pump about as wide as receptacle). Apex of pump with flattened projection. Spermathecal duct long, forming “S” coils.

#### Diagnosis and comparison.

*Distigmoptera chamorrae* can be easily differentiated from all continental speciesof *Distigmoptera* by the bicolorous antennae with antennomeres five in the male and five and six in the female being yellowish, much lighter than the rest of the antennae. It can be distinguished from the only other West Indian species (*Distigmoptera antennata* Medvedev) by the absence of wings (*Distigmoptera antennata* is winged).

#### Etymology.

The specific epithet is a patronym dedicated to Lourdes Chamorro who collected the only known specimens.

#### Type material. 

Holotype: ♂, Puerto Rico: Toro Negro, 18°11.850'N, 66°29.533'W, 904 m, 20.VI.2008, leg. M. L. Chamorro (USNM). Paratype ♀, same label as holotype (USNM).

### Kiskeya

Konstantinov & Chamorro-Lacayo, 2006

http://species-id.net/wiki/Kiskeya

Kiskeya Konstantinov and Chamorro-Lacayo, 2006: 276 (type species *Kiskeya baorucae* Konstantinov and Chamorro-Lacayo, 2006 by original designation, type depository, USNM).

#### Discussion.

Discovery of about a hundred specimens of a third species of *Kiskeya* in Puerto Rico provided additional material that allowed us to observe structures that were not available for study at the time of the description of the genus (Konstantinov and Chamorro-Lacayo 2006).

Labrum 1.54 times as long as wide and 0.57 times longer than thorma (Fig. 31). Labium as long as wide. Apical labial palpomere conical, longer than wide, slightly longer than palpomeres two and three separately (Fig. 30). Mandible with 4 denticles and well developed prostheca (Fig. 33). Pro- and mesotibia flat, widening apically and abruptly narrowing from apical one-third to apex (Figs 35, 36). Metendosternite typical to one of flightless flea beetles with short stalk and relatively long arms with poorly developed tendons (Fig. 34).

**Figures 28–34.**
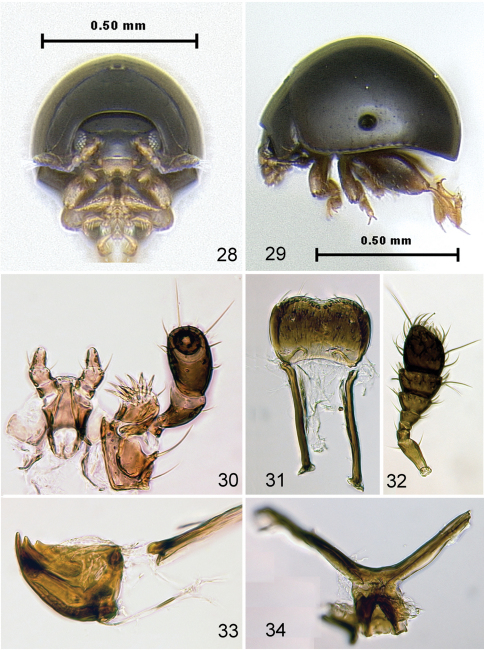
*Kiskeya elyunque*: **28** habitus, frontalview **29** habitus, lateral view **30** labium and maxilla **31** labrum **32** last seven antennomeres **33** mandible **34** metendosternite.

### Kiskeya elyunque

Konstantinov & Konstantinova sp. n.

urn:lsid:zoobank.org:act:77AC612D-625B-4C57-9798-8144A0751C1E

http://species-id.net/wiki/Kiskeya_elyunque

Figs 28 –41

#### Description.

Body length 0.81–0.92 mm, width 0.52–0.65 mm. Color black with light greenish luster. Femur dark brown, rest of legs and antenna dark yellow. Vertex smooth, without punctures or wrinkles. Supraantennal sulcus absent. Pronotum with tiny, sparse, sharp punctures. Antennal club with 3 antennomeres (Fig. 32). Elytron (Fig. 29) convex in lateral view [length (from apex to connection with pronotum) nearly equal to height], with tiny, sparse, barely visible punctures. Proportions of protarsomeres of female (starting with first) 4:2:3:10; mesotarsomeres 4:2:3:10; metatarsomeres 11:2:3:10. In male, proportions as follows: protarsomeres 5:2:3:10; mesotarsomeres 5:2:3:10; metatarsomeres 10:2:2:10. Apex of median lobe of aedeagus without acute denticle in ventral view (Fig. 38). Vaginal palpi with eight long setae posteriorly (Fig. 40), curved towards middle. Anterior sclerotizations widening anteriorly, strongly diverging. Spermatheca (Fig. 39) with receptacle longer and wider than pump. Outer and inner sides of receptacle nearly equally convex. Tignum curved laterally. Posterior sclerotization of tignum wider than middle, arrow shaped (Fig. 41).

#### Etymology.

The specific epithet is a noun in apposition based on the type locality.

#### Diagnosis and comparison.

*Kiskeya elyunque* is the only species of *Kiskeya* known to occur in Puerto Rico. It can be easily differentiated from the other two known species of the genus based on the key below.

#### Ecology.

Unidentified moss samples which contained *Kiskeya elyunque* were collected in the forest from a variety of substrates (rocks, tree stumps, trunks and branches) (Fig. 59, 60).

#### Type material.

Holotype: ♂, Puerto Rico: El Yunque, El Toro trail, 18°11.850’N, 66°29.533'W, 1066m, 14.VI.2008, moss (unsifted) leg. A. Konstantinov (USNM). Paratypes: 45 specimens, same label as holotype (USNM); 37 specimens, same label as holotype except the date, 16.VI.2008 and moss being “unsifted” (USNM).

##### Key to speciesof *Kiskeya*

**Table:** 

1	Supraantennal sulcus present.Apex of median lobe of aedeagus thick and straight in lateral view with narrowly rounded denticle (Fig. 46)	*Kiskeya neibae* Konstantinov & Chamorro-Lacayo
–	Supraantennal sulcus absent	2
2 (1)	Apex of median lobe of aedeagus with acute denticle in ventral view (Fig. 45). Posterior sclerotization of tignum not wider than middle, shapeless (Fig. 44). Internal side of spermathecal receptacle nearly straight (Fig. 42). Posterior ends of vaginal palpi directed laterally (Fig. 43)	*Kiskeya baorucae* Konstantinov & Chamorro-Lacayo
–	Apex of median lobe of aedeagus without acute denticle in ventral view (Fig. 38). Posterior sclerotization of tignum wider than middle, arrow shaped (Fig. 41). Internal side of spermathecal receptacle convex (Fig. 39). Posterior ends of vaginal palpi directed medially (Fig. 40)	*Kiskeya elyunque* sp. n.

**Figures 35–41.**
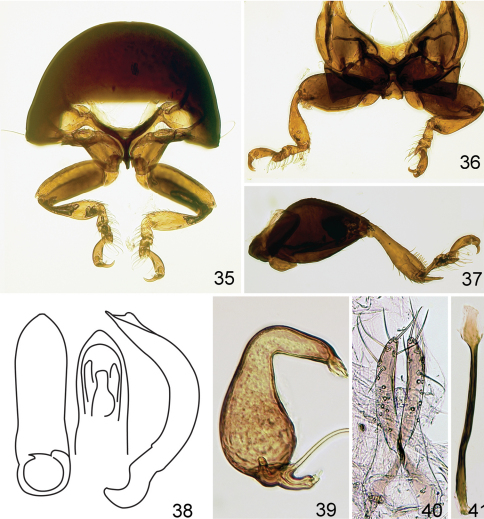
*Kiskeya elyunque*: **35** prothorax, frontal view **36** mesosternum, ventral view **37** hind leg **38** median lobe of aedeagus, ventral, dorsal and lateral views **39** spermatheca **40** vaginal palpi **41** tignum.

**Figures 42–46.**
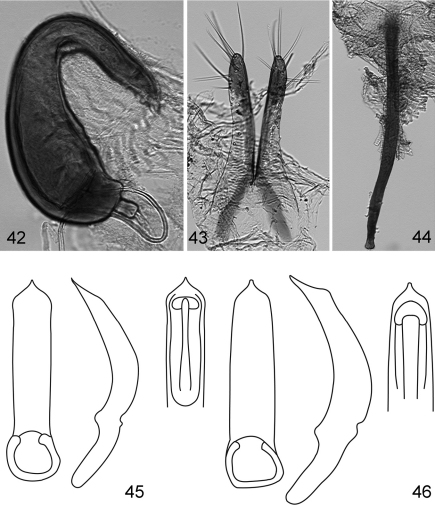
*Kiskeya* species:*Kiskeya baorucae*:**42** spermatheca **43** vaginal palpi **44** tignum **45** *Kiskeya baorucae* median lobe of aedeagus, ventral, lateral and dorsal views **46** *Kiskeya neibae* median lobe of aedeagus, ventral, lateral and dorsal views.

### Ulrica

Scherer, 1962

http://species-id.net/wiki/Ulrica

Ulrica Scherer 1962: 520 (type species *Sparnus minutus* Jacoby by original designation, BMNH).

#### Discussion.

The only three previously described species of *Ulrica* are known from Venezuela. There are about 20 more species that remain undescribed in various collections, but they were also collected from Venezuela. Among West Indian genera, *Ulrica* can be recognized based on the following key. The five specimens of *Ulrica* that were collected in Puerto Rico come from the same mountain region, El Yunque. Based on the labels, the substrate that they came from differs for two species: it is moss for *Ulrica eltoro* and leaf litter for *Ulrica iviei*. The exact collecting locations for *Ulrica iviei*are unknown.

### Ulrica eltoro

Konstantinov & Konstantinova sp. n.

urn:lsid:zoobank.org:act:F5BD9D1D-4F73-4F1B-894B-6640B4C652F1

http://species-id.net/wiki/Ulrica_eltoro

Figs 47–50

#### Description.

**Figures 47–50.**
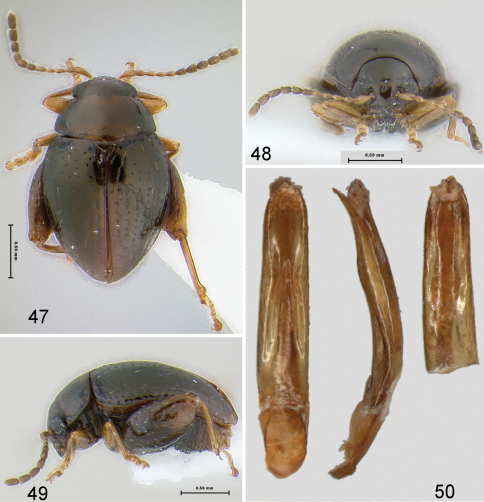
*Ulrica eltoro*: **47** habitus, dorsal view **48** habitus, frontalview **49** habitus, lateral view **50** median lobe of aedeagus, ventral, lateral and dorsal views.

Body length 1.94–2.16 mm, width 1.18–1.29 mm. Color chestnut brown with appendages lighter (Fig. 47). Head surface shiny with few small punctures (Fig. 48), supraorbital pore much larger than a few small punctures on vertex. Supracallinal sulcus clearly separates antennal calli and vertex medially. Frontal ridge wide, longer than antennal calli. Anterofrontal ridge making long denticle about as long as half clypeus length. Pronotum and elytron with fine punctures. Interspaces of elytron flat. Proportions of tarsomeres of male as follows: protarsomeres 7:4:4:9; mesotarsomeres 7:4:4:9; metatarsomeres 10:4:4:9. Median lobe of aedeagus more or less parallel sided in ventral, median lobe view, with ridge in middle being wider at base, narrowing towards middle and widening and disappearing towards apex. In lateral, median lobe view slightly curved with bump on ventral side beyond middle (Fig. 50).

#### Etymology.

The specific epithet is a noun in apposition based on the type locality.

#### Diagnosis and comparison.

*Ulrica eltoro* can be easily differentiated from *Ulrica iviei*based on the key below.

#### Ecology.

Unidentified moss samples that contained *Ulrica eltoro* were collected in the forest from a variety of substrates (rocks, tree stumps, trunks and branches) (Figs 59, 60).

#### Type material.

Holotype: ♂, Puerto Rico: El Yunque, El Toro trail, 18°16.850'N, 65°49.753'W, 1066m, 14.VI.2008, moss (unsifted) leg. A. Konstantinov (USNM). Paratype ♂, same label as holotype (USNM).

### Ulrica iviei

Konstantinov & Konstantinova sp. n.

urn:lsid:zoobank.org:act:1DCF15CA-E09E-4B16-9F13-FBDD84421895

http://species-id.net/wiki/Ulrica_iviei

Figs 51 –58

#### Description.

**Figures 51–55.**
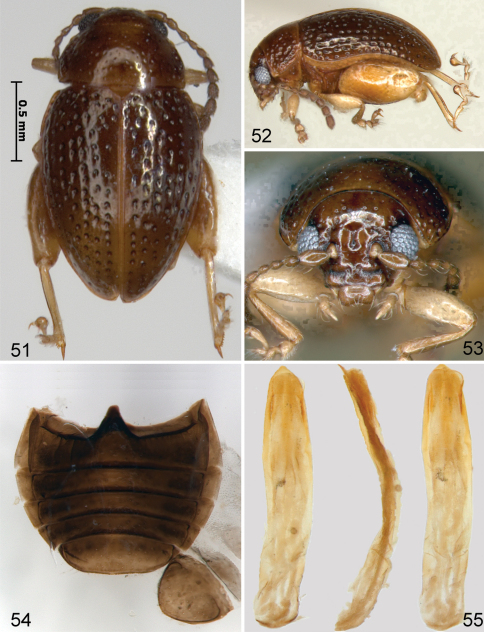
*Ulrica iviei*: **51** habitus, dorsal view **52** habitus, lateral view **53** head, frontalview **54** abdominal sternites, female **55** median lobe of aedeagus, ventral, lateral and dorsal views.

**Figures 56–58.**
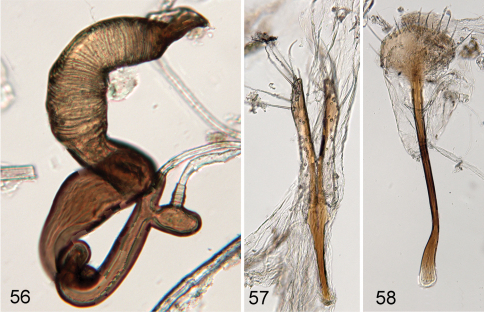
*Ulrica iviei*: **56** spermatheca **57** vaginal palpi **58** tignum.

Body length 1.89–2.05 mm, width 1.08–1.29 mm. Color chestnut brown to almost black with appendages lighter (Figs 51, 52). Head surface dorsally shiny, ventrally with some wrinkles (Fig. 53). Vertex with several large punctures, supraorbital pore as large as a few punctures on vertex near it. Supracallinal sulcus not separating antennal calli and vertex medially. Frontal ridge wide, about as long as antennal calli (Fig. 53). Anterofrontal ridge making long denticle about as long as entire clypeus. Pronotum and elytron with coarse punctures. Interspaces of elytron flat on disc, slightly convex apically. Proportions of tarsomeres of male as follows: protarsomeres 5:4:5:9; mesotarsomeres 5:4:5:9; metatarsomeres 10:5:5:9. Median lobe of aedeagus with more or less curved sides in ventral view, with ridge in middle being wider at base, narrowing towards middle and widening towards apex. In lateral view slightly curved without bump on ventral side beyond middle (Fig. 55). Spermatheca with pump at base wider than receptacle and duct making coils (Fig. 56). Sternite eight nearly fully sclerotized with tignum sharply bent anteriorly (Fig. 58). Vaginal palpi merged at about apical one third (Fig. 57).

#### Etymology.

The specific epithet is a patronym dedicated to Mike Ivie who collected the holotype.

#### Diagnosis and comparison.

*Ulrica iviei*can be easily differentiated from *Ulrica eltoro* based on the key below.

#### Type material.

Holotype: ♂, Puerto Rico: Caribbean Nat. For. Pico El Yunque, El Toro trail, 975 m, 23 Sept. 1987, leg. M. A. Ivie, dwarf forest litter (WIBF). Paratypes ♂ and ♀, Puerto Rico El Yunque, Mt. Britton Tr. VIII.11.1999, C. W. O’Brien, P. Kovarik (MLBU, USNM).

##### Key to species of *Ulrica* from Puerto Rico

**Table:** 

1	Head with supraorbital pore much larger than a few small punctures on vertex. Supracallinal sulcus separating antennal calli and vertex medially. Pronotum and elytron with fine punctures (Fig. 47)	*Ulrica eltoro* sp. n.
–	Head with supraorbital pore as large as a few punctures on vertex near it. Supracallinal sulcus not separating antennal calli and vertex medially. Pronotum and elytron with coarse punctures (Fig. 51)	*Ulrica iviei* sp. n.

**Figures 59–60.**
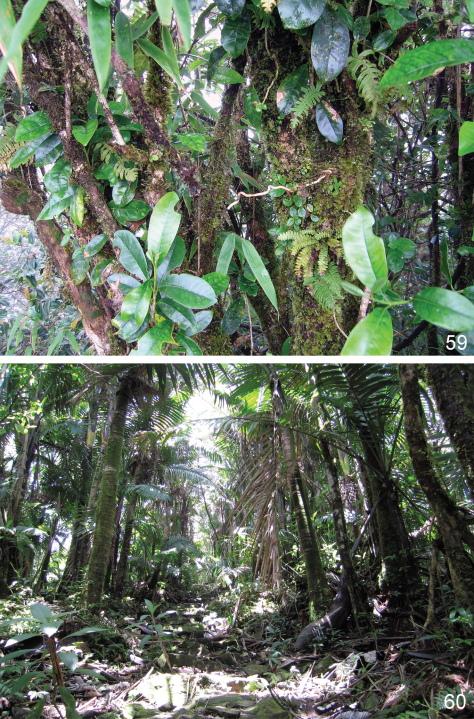
El Yunque, Puerto Rico: **59** moss on tree trunks **60** forest along El Toro trail where moss occurs.

##### Key to Monoplatini genera of the West Indies

In addition to *Distigmoptera* and *Ulrica*, six other Monoplatini genera are reported in the West Indies: *Aedmon* Clark, *Apleuraltica*, *Bonfilsus* Scherer, *Homotyphus* Clark, *Hypolampsis* Clark, and *Physimerus* Clark (Takizawa 2003). *Aedmon*, *Apleuraltica*, and *Bonfilsus* are West Indian endemics, relatively small (with the exception of *Aedmon*) and relatively well circumscribed. However, *Homotyphus* (with about 20 species mostly from South America and just one in the West Indies), *Hypolampsis* (with about 50 species from North, Central, and South America and just two in the West Indies), and *Physimerus* (with about 60 species from Central and South America and one in the West Indies) are poorly understood; their identity, distinguishing characters and composition need extensive review. Below we provide a key for Monoplatini genera of the West Indies based on the West Indian species.

**Table:** 

1	Apical spur of metatibia as long as second metatarsomere. Elytron generally bare, with just a few long setae	*Ulrica*
–	Apical spur of metatibia much shorter than second metatarsomere. Elytron, densely covered with numerous short setae	2
2(1)	Supracallinal sulcus absent, antennal calli merged with vertex	*Bonfilsus*
–	Supracallinal sulcus present, antennal calli separated from vertex	3
3(2)	Antennal calli much longer than wide. Basal antennomeres about same width as apical.	4
–	Antennal calli about as long as wide. Basal antennomeres much narrower than apical	7
4(3)	Pronotal surface more or less even, without two protuberances separated by relatively deep furrow	5
–	Pronotal surface uneven, with two protuberances separated by relatively deep furrow	6
5(4)	Pronotum about as long as wide	*Physimerus*
–	Pronotum significantly wider than long	*Aedmon*
6(4)	Pronotal protuberances very well developed, tall. Two denticles before metatibial apex symmetrically developed	*Homotyphus*
–	Pronotal protuberances poorly developed, low. Two denticles before metatibial apex asymmetrically developed, one on lateral side much wider than one on medial side	*Hypolampsis*
7(3)	Elytron with deep impression posterior to basal callus. Metatibia with transverse ridge above insertion of tarsus. Metatarsal claw in male simple	*Apleuraltica*
–	Elytron without deep impression posterior to basal callus. Metatibia without transverse ridge above insertion of tarsus. Metatarsal claw in male split	*Distigmoptera*

## Supplementary Material

XML Treatment for Borinken

XML Treatment for Borinken elyunque

XML Treatment for Distigmoptera

XML Treatment for Distigmoptera chamorrae

XML Treatment for Kiskeya

XML Treatment for Kiskeya elyunque

XML Treatment for Ulrica

XML Treatment for Ulrica eltoro

XML Treatment for Ulrica iviei
